# Excitation of a Single Compound by Light and Ultrasound Enhanced the Long-Term Cure of Mice Bearing Prostate Tumors

**DOI:** 10.3390/ijms241310624

**Published:** 2023-06-25

**Authors:** Joseph Cacaccio, Farukh A. Durrani, Ishaan Kumar, Mykhaylo Dukh, Susan Camacho, Zahra Fayazi, Adam Sumlin, Eric Kauffman, Khurshid Guru, Ravindra K. Pandey

**Affiliations:** 1 PDT Center, Cell Stress Biology, Roswell Park Comprehensive Cancer Center, Buffalo, NY 14263, USA; joseph.cacaccio@roswellpark.org (J.C.); farukh.durrani@roswellpark.org (F.A.D.); mykhaylo.dukh@roswellpark.org (M.D.); zahra.fayazi@roswellpark.org (Z.F.);; 2 Department of Urology, Roswell Park Comprehensive Cancer Center, Buffalo, NY 14263, USAkhurshid.guru@roswellpark.org (K.G.)

**Keywords:** photodynamic therapy, sonodynamic therapy, combination therapy

## Abstract

Current treatment for prostate cancer is dependent on the stages of the cancer, recurrence, and genetic factors. Treatment varies from active surveillance or watchful waiting to prostatectomy, chemotherapy, and radiation therapy in combination or alone. Although radical prostate cancer therapy reduces the advancement of the disease and its mortality, the increased disease treatment associated morbidity, erectile dysfunction, and incontinence affect the quality of life of cancer survivors. To overcome these problems, photodynamic therapy (PDT) has previously been investigated using Photofrin^TM^ as a photosensitizer (PS). However, Photofrin-PDT has shown limitations in treating prostate cancer due to its limited tumor-specificity and the depth of light penetration at 630 nm (the longest wavelength absorption of Photofrin^TM^). The results presented herein show that this limitation can be solved by using a near infrared (NIR) compound as a photosensitizer (PS) for PDT and the same agent also acts as a sonosensitizer for SDT (using ultrasound to activate the compound). Compared to light, ultrasound has a stronger penetration ability in biological tissues. Exposing the PS (or sonosensitizer) to ultrasound (US) initiates an electron-transfer process with a biological substrate to form radicals and radical ions (type I reaction). In contrast, exposure of the PS to light (PDT) generates singlet oxygen (type II reaction). Therefore, the reactive oxygen species (ROS) produced by SDT and PDT follow two distinct pathways, i.e., type I (oxygen independent) and type II (oxygen dependent), respectively, and results in significantly enhanced destruction of tumor cells. The preliminary in vitro and in vivo results in a PC3 cell line and tumor model indicate that the tumor specificality of the therapeutic agent(s) can be increased by targeting galectin-1 and galectin-3, known for their overexpression in prostate cancer.

## 1. Introduction

Prostate cancer (PCa) is one of the most encountered malignancies, and the second cause of cancer-related deaths among men around the world. African American (AA) men are at an increased risk of developing cancer in comparison to other races [1,2]. Surgery, brachytherapy, and external beam radiotherapy are generally used in clinics for the treatment of localized prostate cancer; however, all these treatments suffer from several side effects, such as incontinence, impotence, and potential damage occurring to other organs and healthy tissues [3]. Therefore, in recent years, there has been growing interest in the development of enhanced tumor-selective treatment for prostate cancer [4]. One of the treatment modalities that has been explored for some time is photodynamic therapy (PDT) [3,4] with limited success, due to (a) the depth of tissue penetration by light [5] and (b) the tumor cell specificity of most of the second-generation photosensitizers (PSs) [6,7].

PDT is a unique form of therapy by which a photochemical reaction between the light and PS in the presence of oxygen generates singlet oxygen with the capability of causing tissue injury and necrosis [7]. However, deeply seated cancerous tissues, especially the prostate, could be difficult to treat with light. Conversely, the depth of tissue penetration by light can be solved by using ultrasound (US) for exciting the photosensitizing agents. The depth of US penetration is usually presented in terms of the half-value depth for the specific ultrasound frequency. Therefore, 1–3 MHz continuous ultrasound, with a half-value depth of approximately 2.3 cm, is frequently used to treat deep tissues that are approximately up to 5 cm deep. The use of US in chemical reactions provides specific activation based on the physical phenomenon of acoustic cavitation [8,9]. Cavitation is a process in which mechanical activation destroys the attractive forces of the molecule in the liquid phase. Applying ultrasound, compression of the liquid is followed by expansion, in which a sudden pressure drops forms small, oscillating bubbles of gaseous substances [10]. The results of SDT alone or in combination with PDT for the treatment of a variety of tumors in animal models and in human patients have been reported by various investigators [11,12]. However, most of the agents evaluated are based on porphyrin and non-porphyrin compounds with long-wavelength absorption below 650 nm [13]. Therefore, in order to take advantage of combination therapy, it is of the utmost importance to select a PS for use in PDT with long wavelength absorption (>660 nm) and enhanced tumor specificity.

It has been reported [13,14] that when PDT was applied to low-risk, moderately differentiated prostate cancer patients, less than a third of the patients had clinically apparent disease. On the other hand, patients with poor differentiation or those who were at high risk showed a reduction of 42% of the tumors, but the total PSA level was still higher than 10 ng/mL, suggesting that PDT alone is not suitable for these patients. Additionally, recent advances in PDT have allowed for specific targeting of the vascular system that feeds the tumor [14,15]. In one clinical trial, 24 patients were enrolled in a phase I/II trial to investigate the potential of avascular treatment; 6 months after treatment, there was no tumor mass observed by MRI [16,17]. These preliminary clinical data demonstrate the potential of PDT in treating a wide variety of prostate cancer phenotypes. Our own findings in animal models and cancer patients also suggest that HPPH [3-1′(hexyloxy)ethyl-3-devinylpyropheophorbide-a] [18,19,20,21] is an effective PDT agent for treating a variety of cancer patients [22,23,24], which in combination with SDT shows improved treatment outcomes for brain cancer (glioblastoma) [17]. It was observed that the reduction in the PS concentration in tumors by light exposure due to photobleaching upon further exposure to US causes dilation of tumor vasculature, resulting in a significant increase in circulating PS in tumors, and enhances tumor destruction due to increased ROS formation and independent of the oxygen concentration in the tumor [17].

Similar to whole-body hyperthermia (WBH), the increase in localized intertumoral temperature by SDT may boost the infiltration of CD8^*^ T cells into the tumor microenvironment, benefiting patients’ survival in the long term [24]. WBH has been shown to increase the number of perfused blood vessels within a tumor, allowing for a significant increase in radio sensitivity and chemo sensitivity [25]. It is also known to lead to a decrease in tumor interstitial fluid pressure and enhanced tumor perfusion, resulting in a significant decrease in hypoxia within the tumor microenvironment, with improved-tumor re-oxygenation and ROS production. These changes might help in augmenting the effect of PDT both through higher tumor cell kill and increasing anti-tumor response.

The combination of localized heat generated by high-dose ultrasound without a PS showed a significant increase in the long-term response to treatment and survival of cancer patients [26]. Diarrhea, nausea, and vomiting are some of the common side effects of high-intensity focused ultrasound (HIFU), which can possibly be prevented with localized SDT using lower doses of ultrasound in combination with PDT to provide enhanced long-term cure for prostate cancer patients.

In recent years, a variety of PSs have been reported. The synthetic design is focused to improve tumor specificity by targeting those receptors known for their high expression in various tumor types [27,28,29]. Among the targets, galectin-3 (Gal-3) is of particular interest for developing prostate cancer-specific agents [30,31,32]. It is a member of a family of proteins that contain at least one β-galactoside-binding protein. These proteins are associated with growth and early embryonic development. In adult cells, Gal-3 is found primarily in epithelial and myeloid cells in the cytoplasm [33]. Once Gal-3 is exported to the extracellular matrix, it forms oligomeric structures that interact with a wide variety of substrates. These interactions can induce signals between two different cells, the cell, and the extracellular matrix or even induce a signal within the same cell. Galectin-3 specifically is an attractive target for cancer research because it is not expressed in high amounts in the body beyond early development. Additionally, the structure of Gal-3 and its binding affinity to various carbohydrates (β-galactose analogs) is well known and has been studied for a long time [33]. For example, Young Park et al. [34] utilized nanotubes conjugated with β-galactose to detect Gal-3 in the blood of cancer patients and used it as a biomarker of cancer. Additionally, Balakrishnan et al. [35] utilized gal nanoparticles to specifically target breast cancer and demonstrated a 30-fold increase in doxorubicin delivery to the tumor. Another study used the Gal-3 targeting protein G3-H12 to target HPMA particles in prostate cancer [36].

We have previously shown that conjugating β-galactose with a photosensitizer derived from chlorophyll-a (e.g., HPPH analogs) enhances its photosensitizing efficacy in tumors with high expression of Gal-3 [37,38,39]. We extended this approach by synthesizing a series of HPPH–galactose conjugates in which the carbohydrate moiety was conjugated either at 17 or 20 or both of the positions of HPPH [39,40]. The best conjugate from this series was further tested for uptake and retention as well as synergistic interaction of PDT +SDT to illustrate the benefit of gal-3 targeting in tumor uptake and efficacy.

The current study is focused on comparing the in vitro PDT efficacy of HPPH and the corresponding carbohydrate analogs and identifying the best candidate for the treatment of prostate cancer by using a combination of PDT and SDT.

## 2. Results and Discussions

**Figure 1:**
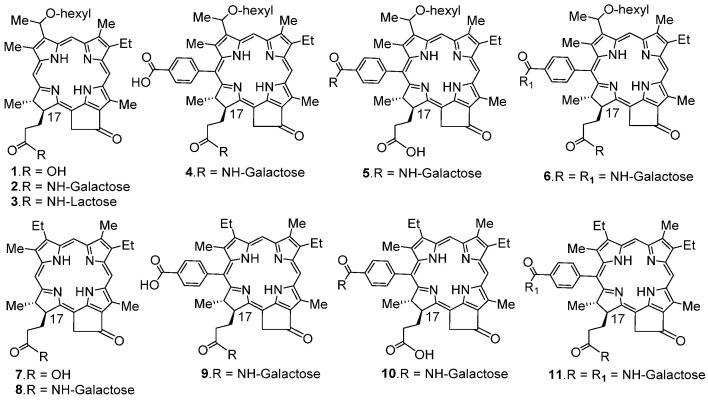
Structures of photosensitizer-carbohydrate conjugates investigated in current study.

**Chemistry:** HPPH and the corresponding 17 and/or 20 β-galactose analogs (with and without a hexyl ether side chain at position-3) were synthesized by following our own methodology [39,40]. The structures of the compound are shown in Figure 1.

### Biological Studies

**Figure 2:**
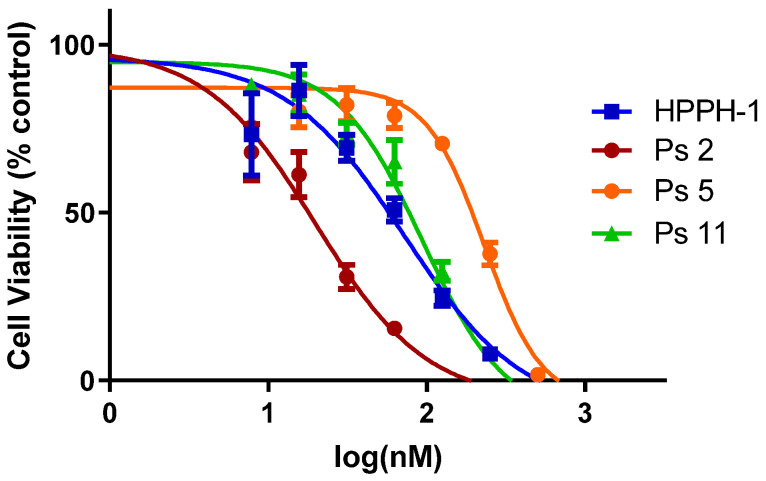
Comparative in vitro PDT efficacy (% cell survival) of HPPH 1 with selected b-galactose analogs. Among these probes, HPPH–Gal 2 showed the best in vitro efficacy (light dose: 1 J/cm^2^). See the text and Table 1.

**Figure 3:**
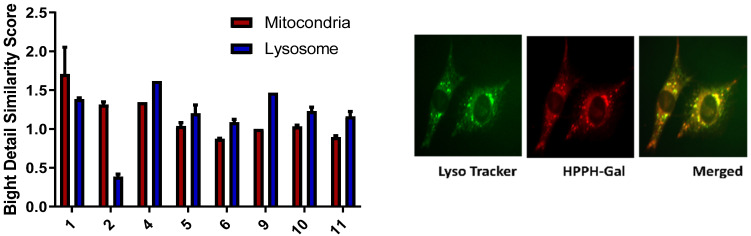
Comparative intracellular localization (mitochondria vs. lysosomes) of PSs with and without the b-galactose moiety.

(a) *In vitro photosensitizing efficacy:* The in vitro data suggest that the addition of carbohydrate moieties to HPPH changes the biological activity in multiple ways; one of the most immediate and evident changes to the biological function of these derivatives is the change in overall lipophilicity. This in part governs the uptake, retention, and localization of the molecule which can substantially alter the PDT efficacy. To evaluate the in vitro efficacy of these compounds, the PSs were incubated in PC3 cells (known for overexpression of Gal-3) for 24 h and the photosensitizing efficacy of each PS was determined by the MTT assay. The IC_50_ values of HPPH were compared with a series of photosensitizers. The results summarized in Table 1 and Figure 2 indicate that among all the compounds, the HPPH conjugated with a b-galactose moiety at position 17^2^- showed the best efficacy.

(b) *Subcellular localization of HPPH and the corresponding carbohydrate analogs:* The subcellular localization of a PS is an important property that is highly dependent on its chemical structure [41]. Previous studies have suggested that mitochondrial vs. lysosomal localization leads to two very different cell death pathways: localization of the PS in the mitochondria leads to cytochrome release and photoinduced necroptosis, while targeting the lysosome has been connected to autophagy [42]. To determine how galactose conjugation changes how HPPH localizes in the cell, the cells were visualized using an Amis Imagestream Mkit. HPPH has previously been shown to have a slight preference for mitochondrial localization in other cell lines, but in PC3 cell lines, there is no preference. The addition of a galactose moiety to HPPH either at the 17 or 20 position alters the localization dramatically. This observation is possibly due to Gal-3 receptor-mediated endocytosis and lysosomal localization. The subcellular localization of the representative galactose-conjugate **2** follows a similar distribution of HPPH, preferring the mitochondria over the lysosome. The mitochondrial bright detail similarity score observed from **2** was three times higher than the lysosomal detail similarity score. On the other hand, the HPPH mitochondrial bright detail similarity score was only 1.5 times greater than the lysosomal bright detail similarity score. Meanwhile, **PS 11**, the other galactose-conjugated photosensitizer which showed a high degree of PDT efficacy, slightly prefers the lysosome over the mitochondria (Figure 3). A similar trend was also observed in the other carbohydrate analogs.

In the case of the carbohydrate conjugates, initial binding was observed at the cell surface followed by internalization, and progressive lysosomal accumulation was evident, and it became more prominent at 24 h post incubation, probably in part due to sequestration into the organelle. The change in the site of localization from the mitochondria to the lysosomes suggests that between the HPPH and the corresponding carbohydrate conjugates, HPPH was subjected to a different mode of uptake and intracellular distribution. The predominance of HPPH–Gal in the lysosomes could be due to the involvement of endocytosis, i.e., through phagocytosis/pinocytosis.

Finally, to investigate the uptake kinetics and target-specificity of the Gal–PS conjugates to Gal-3, a series of experiments were performed using a Zeiss microscope. In the first set of experiments, the uptake of the individual PSs was determined by incubating them in PC3 cells for 4 h. In the second set of experiments, the PC3 cells were incubated with free β-Galactose in excess along with the PS, and its uptake was observed at 4 h. As expected, the cell uptake of HPPH was not affected by the addition of β-Galactose, whereas, in most of the carbohydrate conjugates, it was significantly reduced due to the presence of β-galactose, suggesting a target-specificity of these PSs to the Gal-3 receptor. While Ps **2** exhibited the most inhibition in cell uptake by the competitive assay among the other carbohydrate analogs, the presence of an ethyl vs. a hexyl ether side chain at position 3 also made a significant difference in the uptake/retention of the PS in tumor cells.

**Figure 4:**
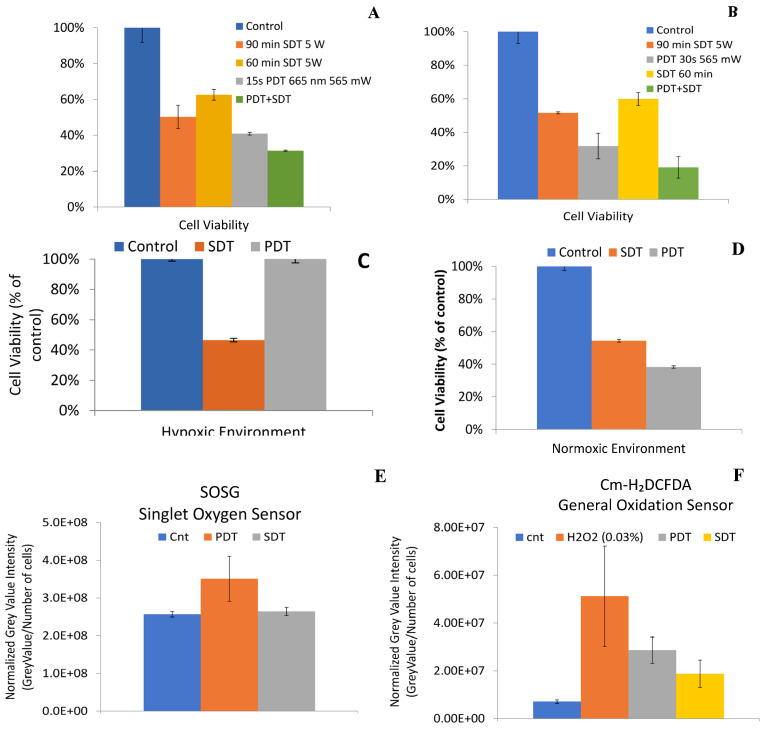
In vitro PDT and SDT efficacy of HPPH in the PC3 cells under hypoxic and non-hypoxic conditions. HPPH SDT efficacy in combination with PDT (**A**) demonstrates a significant decrease in viability over either modality alone, (**B**) compound 2 shows an even greater improvement in cell kill by combining PDT with SDT, (**C**,**D**) demonstrate HPPH cell kill potential in both hypoxic and normoxic environments, (**E**) SDT efficacy is not induced via singlet oxygen, and (**F**) SDT produces mainly radical ions (type I mechanism for the formation of reactive oxygen species). The PSs were used in an equimolar concentration (1 μM).

**Figure 5:**
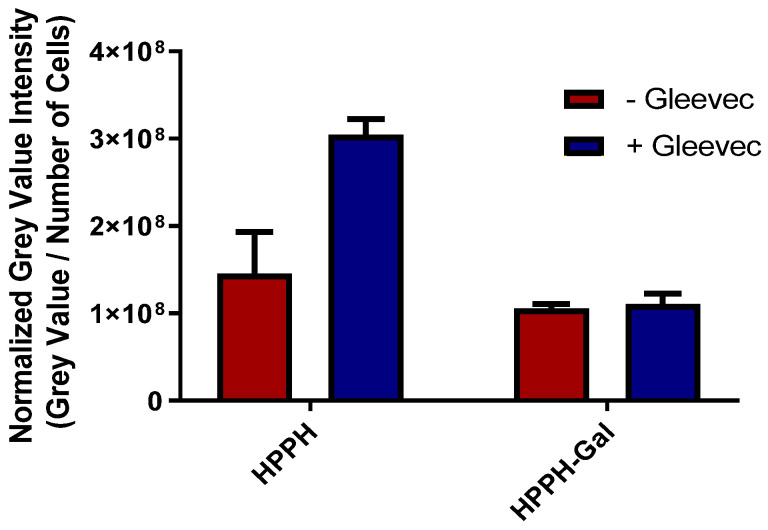
Intracellular uptake of HPPH and HPPH–Gal with and without the presence of Gleevec, a tyrosine kinase inhibitor.

(c) *Mechanisms of PDT and SDT in tumor cell kill:* Sonodynamic therapy is a promising addition to photodynamic therapy which utilizes a different method of generating cell kill and subcellular damage [43]. Compound **2** was tested in the PC3 cells to determine the potential of PDT in combination with SDT. Both HPPH and compound **2** showed enhanced cell-killing efficacy when both treatment modalities are used in combination (Figure 4A,B). It is well established that PSs generate singlet oxygen species during exposure to an appropriate wavelength of light, which is responsible for inducing the cytotoxic effect. To determine if SDT is equally dependent on oxygen and utilizes singlet oxygen to induce cell kill, the reactive oxygen species (ROS) generated after exposing the compound used for PDT by US was identified in vitro using either carboxy-DCFDA (Figure 4C,D) or SOSG (singlet oxygen sensor green) for singlet oxygen (Figure 4E,F). Caboxy-DCFDA is a general indicator of ROS, while SOSG is a dye sensitive to only singlet oxygen [44]. The PC3 cells were then exposed to ultrasound and the fluorescence observed from the cells clearly indicates that the radical ions were formed by exciting the compound with ultrasound. The experimental result revealed that HPPH and compound **2** have similar efficacy when PDT was combined with SDT treatment.

(d) *SDT and hypoxia:* The utility of SDT for treating cancer cells under a hypoxic environment was further confirmed. In brief, the PC3 cells were incubated with HPPH and PS **2** (1 μM). After 24 h incubation, some of the plates were placed in a hypoxic chamber at 1% O_2_ and 5% CO_2_ for 1 h to deplete the oxygen. The cells were then exposed to either light only or US only. At 24 h after exposure, the cells were counted using a trypan blue assay. As expected, light exposure (PDT) did not induce cell death due to the lack of oxygen needed to generate singlet oxygen, whereas US exposure produced significant cell kill mainly by radical ions (Type I reaction). In the control experiments, the cells were incubated with PS alone and were not exposed to light or US. The results depicted in Figure 4 show the advantage of SDT in treating hypoxic cells.
(e) *In contrast to HPPH 1, HPPH–Gal 2 is not transported from PC3 cells by ABCG2:* The ABCG2 protein is known to export many photosensitizers from tumor cells and has a significant negative impact on intracellular accumulation. Jonker et al. [45] showed that ABCG2 knock-out mice were photosensitive because of increased protoporphyrin IX (PP IX) levels. Robey et al. [46] reported that pheophorbide-a and its methyl ester analogs are a specific substrate for ABCG2. The results from our laboratory have shown that HPPH (hexyl ether derivative of pyropheophorbide-a) is a substrate of ABCG2, and the presence of a certain tyrosine kinase inhibitor (Gleevec) enhances its PDT efficacy [47]. We extended this approach in the PC3 tumor cell line and observed that Gleevec enhances the uptake of HPPH by inhibiting efflux from the cell. Interestingly, the addition of Gleevec did not have a significant impact on the accumulation of conjugate **2** (Figure 5), which suggests that HPPH–galactose conjugates are not a target of ABCG2 efflux, thus having higher clinical PDT efficacy [48].

In addition to ABCG2 expression, hypoxia is quite common in tumors, especially after PDT due to the depletion of oxygen. Therefore, repeated PDT may have limited benefit in tumor cure. However, the use of SDT should be advantageous as it does not require oxygen to produce ROS (mainly radical ions) for the destruction of tumor cells.

**Figure 6:**
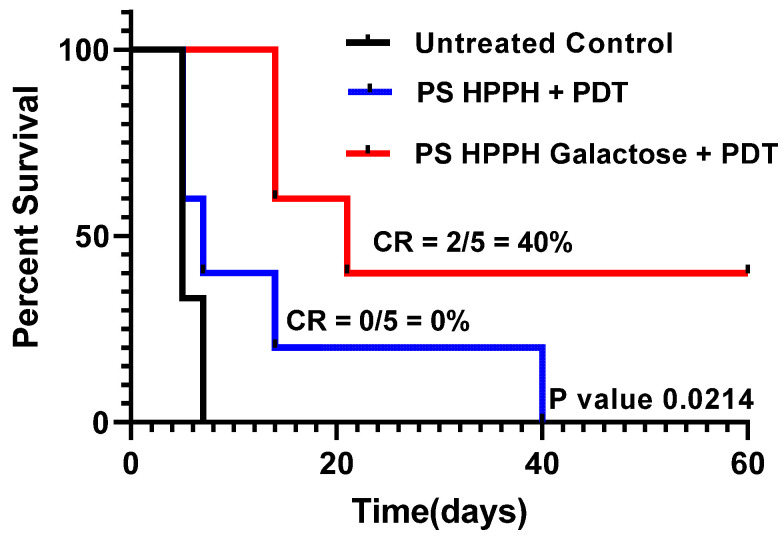
Comparative in vivo PDT efficacy of HPPH 1 vs. HPPH–Gal conjugate 2 under similar treatment parameters in SCID mice bearing PC3 tumor xenografts. For details, see the text.

**Figure 7:**
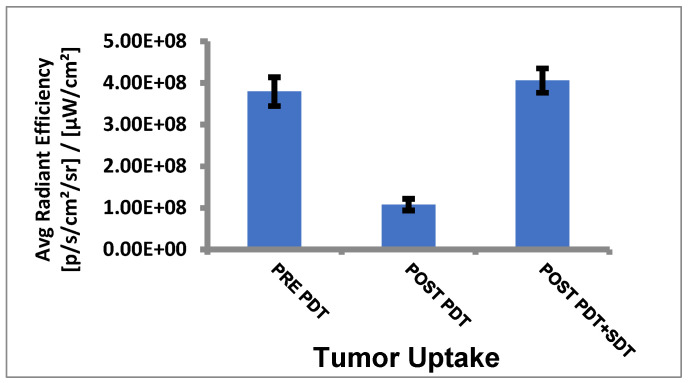
Tumor (PC3) uptake (determined by fluorescence (excitation wavelength: 665 nm; emission wavelength: 720 nm) of HPPH (0.47 µmol/kg) at 24 h pre PDT, post PDT, and then at 24 h SDT in SCID mice (3 mice/group). Light dose: 135 J/cm^2^, 75 mW/cm^2^, for 30 min. US dose: 2 W/cm^2^ for 30 min.

**Figure 8:**
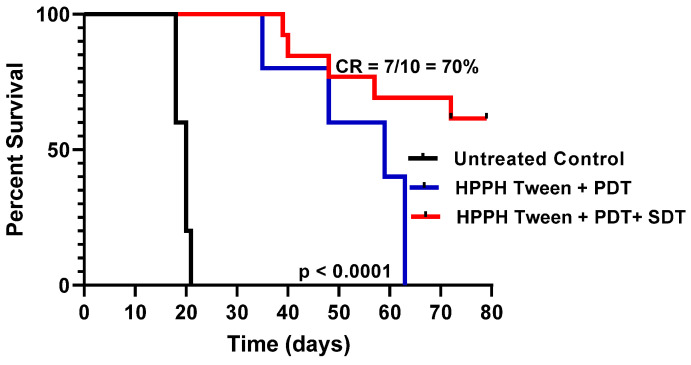
In the first set of experiments, the SCID mice (5 mice//group) bearing PC3 tumors (200–250 mm^3^) at ~day 7 were intravenously injected with HPPH (dose: 0.47 mmole/kg). At 24 h post injection, the tumors were exposed to light (665 nm, 135 J/cm^2^, 75 mW/cm^2^) and tumor growth was monitored everyday (shown in the solid blue line). In the second set of experiments, the SCID mice (5 mice with tumors/group) were first treated with light as discussed above, and then at 24 h after PDT treatment, the tumors were exposed to ultrasound (2 W/cm^2^ for 30 min), and the growth of the tumor in each mouse was monitored daily (shown in the solid red line). The combination approach enhanced the long-term tumor response (7/10 mice were tumor-free at day 80). HPPH-PDT alone was also effective, but no tumor cure was obtained at day 65.

(f) *Compared to HPPH 1, HPPH–Galactose 2 showed improved PDT efficacy in vivo:* The PDT efficacy of both the PSs was investigated in SCID mice bearing PC3 tumor xenografts. When the tumor was around 200–250 mm^3^ in size, each PS was injected at a dose of 0.47 mmole/kg, and at 24 h post injection, the tumors were exposed to light (135 J/cm^2^; 75 mW/cm^2^) (two sets of experiments; five mice/group). Tumor growth was monitored daily. The results summarized in Figure 6 indicate that the tumor regrew in all mice treated with HPPH-PDT at day 60, and the mice were euthanized. Under similar treatment parameters, HPPG–Gal 2 showed improved long-term cure, and 2/5 mice were tumor-free on day 60. These results suggest that the presence of a galactose moiety at the appropriate position of the macrocycle and also the overall lipophilicity maintained by introducing a hexyl ether side chain at position 3 made a significant difference to the biological efficacy in vivo. This was a proof-of-principle study, and the treatment parameters are currently being optimized.

(g) *Tumor uptake of HPPH before and after PDT/SDT in mice bearing PC3 tumors:* The mice were imaged for HPPH, and uptake was determined by measuring the fluorescence intensity in the tumors by using an IVIS system (Perkin Elmer) at various time intervals after injecting the HPPH (dose: 0.47 μmole/kg) into the SCID mice bearing PC3 tumors (3 mice/group) at 24 h (pre PDT), post PDT, and post PDT–SDT treatment. Images (fluorescence) were collected at various time intervals, and the average radiant efficiency of HPPH in the tumors was determined. The results summarized in Figure 7 show a significant uptake of HPPH at 24 h post PS administration. After light treatment, a significant amount of HPPH photobleaches, resulting in reduced fluorescence intensity. However, after irradiating the tumor with ultrasound (US), the concentration of HPPH in the tumor increases, which was surprising and interesting as well. These results suggest that (a) exposure of tumors to US after light treatment further dilates the intratumoral blood vessels, which possibly allows more uptake of the PS from circulation, and subsequently enhances the fluorescence intensity of the PS in the tumor, and (b) US exposure did not photobleach the PS.

(h) *PDT in combination with SDT showed enhanced PDT efficacy:* HPPH **1** formulated in 1% Tween80 was intravenously injected into PC3 tumor-bearing mice at a dose of 0.47 μmol/kg. The drug dose (0.47 mmol/kg) was selected based on our previous study with HPPH in other tumor types, where it showed moderate long-term anticancer activity. For our initial study, PC3 tumors were implanted into SCID mice (5 mice/group), and when the tumor size was around 200–250 mm^3^, the PS was intravenously injected. At 24 h post injection, the tumors were exposed to light (135 J/cm^2^, 75 mW/cm^2^), and tumor growth was monitored. In another set of experiments, a group of SCID mice bearing PC-3 tumors were treated with light alone (control). After PDT treatment, these tumors were exposed to ultrasound (US dose: 2 W/cm^2^ for 30 min), and tumor regrowth was measured three times a week following the IACUC-approved animal protocol. Once the mice reached a tumor > 400 mm^3^, they were euthanized. The results illustrated in Figure 8 show a significantly improved long-term tumor cure of mice with a combination of PDT + SDT treatment over PDT alone.

**Figure 9:**
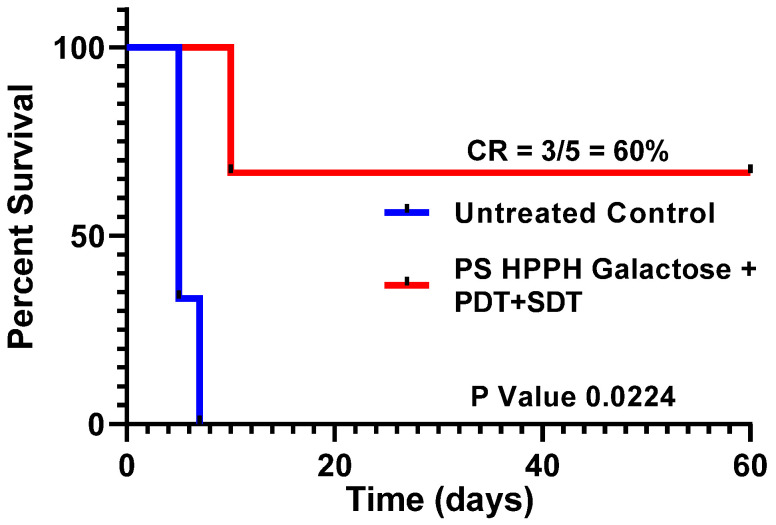
HPPH–Gal 2 was intravenously injected into the SCID mice (5 mice/group) bearing PC3 tumors (subcutaneously implanted at ~day 7) at a dose of 0.47 μmole/kg, and at 24 h post injection, the tumors were exposed to light at a dose of 135 J/cm^2^, 75 mW/cm^2^ (PDT), and at 24 h post PDT, the tumors were exposed to ultrasound (2 W/cm^2^) for 30 min. Tumor growth was measured daily. On day 60, 3/5 mice were tumor free.

In a second study, HPPH–Gal **2**, which showed improved long-term tumor cure over HPPH in SCID mice bearing PC3 tumors, was further evaluated in the PC3 tumor model for PDT + SDT combination therapy using a small number of mice with tumors (a proof-of-principle study), and it also showed improved PDT efficacy (long-term tumor cure, Figure 9). However, in both experiments, the PDT and STD treatment parameters need to be optimized, and these studies are currently underway.

**Figure 10:**
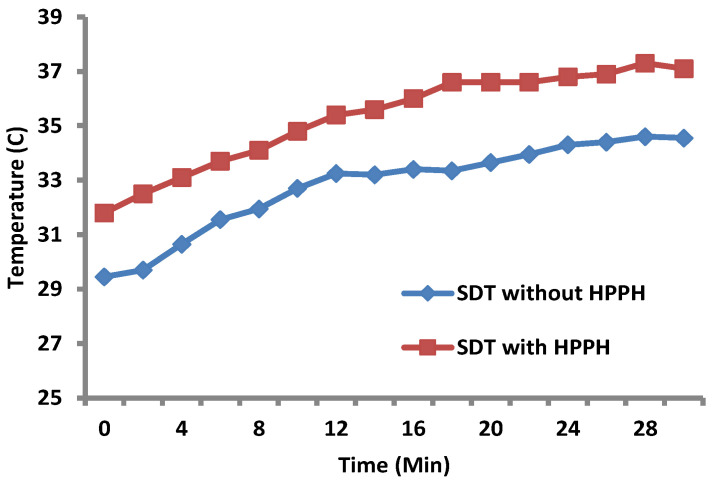
SDT treatment of PC3-tumor-bearing mice (two sets of experiments) with and without administration of HPPH showed a 5–6 °C increase in tumor temperature.

(i) *Increase in tumor temperature (hyperthermia) during SDT:* HPPH **1** at a dose of 0.47 µm/kg was injected into the tail vein of a PC3 = tumor-bearing mouse at 24 h prior to SDT. Sonodynamic therapy was carried out at the ultrasound dose of 3 MHz for 30 min with the temperature probe inserted directly into the tumor. In the control mice, the same procedure was followed except no HPPH in 1% Tween formulation was injected prior to SDT. The results shown in Figure 10 indicate an increase of 4–5 °C in tumor temperature on exposing the tumors with and without the presence of the PS (HPPH). The intra-tumoral temperature in mouse 1 (US alone) increased from 29 to 34 °C and in mouse 2 (US + HPPH), it increased from 31 to 37 °C.

## 3. Methods and Materials

### 3.1. Chemistry

The HPPH and the corresponding carbohydrate analogs were synthesized by following our own methodology [39,40].

### 3.2. In Vitro Cell Culture

The PC3 cells were acquired from ATCC and grown in RPMI supplemented with 10% FBS and 10% streptomycin and penicillin.

### 3.3. In Vitro Viability Assay of Photodynamic Therapy in Combination with Sonodynamic Therapy

To determine the PDT efficacy of the PSs, an MTT assay was performed at 48 h post light irradiation. Briefly, the cells were plated at 1000 cells/mL in 96-well plates. After the cells adhered, the cells were incubated with each photosensitizer for 24 h before being exposed to a 665 nm diode at 565 mW/1 J/cm^2^. MTT was added 48 h post PDT and the absorbance was read in DMSO 3 h post the addition of MTT.

To evaluate the in vitro efficacy of sonodynamic therapy, the cells were counted with trypan blue and compared to a control plate. Each cell plate was exposed to 3 mHz of ultrasound at 5 W. Approximately 24 h after ultrasound exposure, the cells were harvested with trypsin and counted using a hemocytometer. Plates exposed to combination therapy received 15 s of 665 nm light at 565 mW and were then exposed to ultrasound.

### 3.4. Intracellular Localization

To visualize the intracellular localization of each compound within a cell, an Amis Imagestream Mkit was used as previously described. Briefly, the cells were plated in a 6-well plate and allowed to adhere for 24 h. Once the cells adhered to the plastic, 1 µM of the photosensitizer was added to the cells and incubated for 24 h. Before the cells were harvested, Mitotracker Cmxros red (to stain the mitochondria) and Lysosensor gren dnd 189 (to stain the lysosome) were added. The data were analyzed using IDEAS software (Version 6.2) to determine the bright detail similarity score.

### 3.5. Determination of Reactive Oxygen Species

Two dyes were used to determine reactive oxygen species generation in vitro. CM-DCFDA was used to determine general ROS production within the cell as a result of PDT or SDT. The cells were plated in 6-well plates and allowed to adhere. Approximately 5 µM of the photosensitizer was used. Approximately 2 h before PDT or SDT, the cells were washed with PBS, and serum-free media were added with 10 µM of Cm-DCFDA added. The cells were then washed to remove excess DCFDA and exposed to either PDT or SDT. After ROS induction, positive control H_2_O_2_ was added to 0.03%. The plates were immediately imaged using a fluorescent microscope. The results were graphed comparing the fluorescence per cell in the test wells to a negative background control.

To distinguish between type I and type II ROS production, a singlet oxygen sensor green was used to directly detect singlet oxygen in vitro. The cells were plated in 6-well plates, and 5 uM of the photosensitizer was added. Approximately 24 h after PS addition, the cells were washed 3×, and the media were replaced with serum-free media supplemented with 10 uM SOSG. The cells were incubated with SOSG for 2 h before being washed again and fresh media added. The plates were exposed to PDT or SDT and imaged using a fluorescent microscope.

### 3.6. Method for Tumor Implantation

The PC3 tumors were transplanted into 6–8-week-old female SCID mice (Strain C.B Igh-1b Icr Tac Prkdc Scid) and were maintained in the animal facility. Mice bearing an established tumor (~7 days after implantation) were treated with PDT/SDT. Two axes (mm) of tumor (L, longest axis; W, shortest axis) were measured with the aid of a Vernier caliper. Tumor volume (mm^3^) was estimated using a formula: tumor volume  =  ½ (L  ×  W^2^). Complete tumor regression (CR) was defined as the inability to detect the tumor by palpation at the initial site of tumor appearance for more than 2 months post therapy. Partial tumor regression (PR) was defined as a ≥ 50% reduction in initial tumor size. Observations of edema, erythema, and scar formation in the treatment field were observed and recorded. All studies were performed in accordance with protocols approved by the institutional animal care and use committee of our institution (Roswell Park Comprehensive Cancer Center).

### 3.7. Determination of In Vivo PDT and SDT Efficacy

SCID^prkde^ mice with subcutaneous PC3 xenografts of 200–250 mm^3^ were injected intravenously (i.v) with HPPH at a dose of 0.47 μmol/kg (the HPPH dose was selected based on our previous in vivo studies of HPPH-PDT in other cancer types). The PS uptake in PC3 tumors was determined by fluorescence imaging using a PerkinElmer IVIS Spectrum at variable time points, and the maximum uptake was observed at 24 h post injection. Therefore, at this time point, the tumors were irradiated with light (fluence: 135 J/cm^2^; fluence rate: 75 mW/cm^2^) for 30 min at 665 nm using a Lightwave laser diode. The mice were restrained without anesthesia in plexiglass holders designed to expose only the tumor and a 2–4 mm annular margin of skin to light. For SDT exposure, a continuous US dose of power with ultrasound (2 W/cm^2^) for 30 min was administered as described above. For the first 10 days, tumor measurements were taken daily, then three times a week for 4 weeks, and finally twice a week thereafter for a total of 60 days post treatment. Tumor response for each treatment was compared to tumor-bearing animals not subjected to therapy (PDT, SDT, or PDT  +  SDT). The optimization of PS, light, and US doses is currently underway for evaluating other tumor types.

## 4. Conclusions

The results presented herein suggest that conjugation of HPPH with a β-galactose moiety at position 17^2^ is a potential target for galectins (galectin-3 and galectin-1). The conjugate showed improved PDT efficacy in vitro (PC3 cell culture) and in vivo (mice bearing PC3 tumors). PDT in combination with SDT gave synergistic effects and produced enhanced tumor cure. Furthermore, the results presented herein demonstrate that, unlike HPPH–Gal, HPPH is a substrate for ABCG2 in the PC3 cell line. The HPPH–Gal with inhibited transport showed improved therapeutic potential. The combination treatment for prostate cancer with targeted NIR PDT and SDT should reduce the shortcomings of current therapies for prostate cancer, e.g., the risk of urinary incontinence, erectile dysfunction, ejaculation problems, and sexual impotence. Unlike chemotherapy, bone marrow remains intact in PDT/SDT treatment, which should also help in improved tumor cure and provide a better quality of life to cancer patients. In contrast to PDT, SDT is oxygen independent and should be beneficial in treating hypoxic tumors, whereas PDT alone shows limitations.

**Statistical Analysis:** The standard log-rank test (Mantel–Cox) was used for statistical analysis. It is a hypothesis test to compare the survival of the animal/tumor cure based on the Kaplan–Meier survival curve. It is a test significant for detecting differences between groups to confirm if one group has a risk of an event greater than the other. For analysis of the in vivo PDT/SDT efficacy cure rate, the survival curves were plotted using the drug dose over tumor regrowth. The in vivo experiments discussed in this manuscript were performed in compliance with all state, local, and federal laws and the PHS Policy on the Human Care and Use of Laboratory Animals. This study was conducted in an AAALAC-accredited facility, following the IACUC-approved animal protocol.

## Figures and Tables

**Table 1:** Comparative PDT efficacy (IC50 values) of PSs with and without b-galactose conjugates (mono- and di-galactose moieties) with variable lipophilicity.

Compound	PC3 Cell Line (IC_50_ Value)	Log *p*
**HPPH-1**	54 ± 9 nM	6.01
**2**	18 ± 3 nM	5.04
**3**	85 ± 10 nM	5.04
**4**	316 ± 32 nM	4.53
**5**	156 ± 37 nM	4.05
**6**	311 ± 54 nM	2.14
**9**	101 ± 13 nM	3.14
**10**	128 ± 14 nM	3.14
**11**	71 ± 10 nM	1.7

## Data Availability

Not applicable.

## Abbreviations

PS: photosensitizers; PDT: photodynamic therapy; SDT: sonodynamic therapy; HIFU: high-intensity ultrasound; NIR: near infrared.
